# Tuberculosis

**Published:** 1891-07

**Authors:** R. W. Stewart

**Affiliations:** Pittsburg


### TUBERCULOSIS.*

BY R. W. STEWART, M. D., PITTSBURG.

Tuberculosis may be truly said to be the pest of humanity. Few diseases are so insidious in their onset, and none so disastrous in their results. One-seventh of the human race is destroyed by it.

What tuberculosis is, the lesions it produces and the best means with which to combat it, will be my object to describe in this brief paper. Nine years ago Robert Koch proclaimed to the scientific world, and conclusively proved, that tuberculosis was the result of the growth in the tissues of a micro-organism called the bacillus of tubercle. So conclusive has been the proof of Koch's assertions, so thoroughly has all opposition been swept away, that it would be reprehensible on my part to needlessly occupy your time with a history of the discovery of the bacillus of tubercle, and the steady advancement of the views originally promulgated by Koch until all opposition was silenced and antagonism turned to enthusiastic support.

The manner in which the tubercle bacilli gain entrance to the system are various, the respiratory and alimentary tracts being the most common seats of entrance, but there is abundant testimony to prove that abrasions in the skin may afford a vulnerable point for the entrance of the bacilli, as is demonstrated in the production of lupus.

The bacilli may remain localized at the seat of entrance, or circulating in the blood current may fail to find a suitable location for their growth, and be ultimately destroyed or removed with the excretions. A less favorable termination, however, frequently takes place. The bacilli may find a nidus where the conditions are favorable for their growth in almost any of the tissues, but most frequently in the lungs, where they produce the disease called consumption; in the lymphatic glands, where

*Read before the Allegheny County Medical Society, May 19, 1891.

they produce the disease called scrofula, a term now rendered obsolete; in the skin, where it produces lupus; in the bones, where they are said to produce twenty-five per cent. of all chronic inflammatory diseases. The joint structures, the tendons and tendon sheaths, the peritoneum and the brain are frequently affected. In fact, but few of the tissues are exempt from their inroads.

In acute miliary tuberculosis almost all the organs of the body become simultaneously affected. This seldom or never occurs from a primary infection, but nearly always from the absorption of some caseous material from some pre-existing tubercular focus, by which a quantity of the bacilli or their spores are liberated in the blood and attack the tissues, whose vitality is already impaired, and hence rendered less resisting by the presence of a pre-existing disease. So varied are the manifestations and treatment of tuberculosis, according to the tissues affected, that it would be impossible in a paper of this character to do the subject justice. I have, therefore, chosen to confine my remarks as closely as possible to the condition known as pulmonary tuberculosis, because it is the one with which, as a body, we are most familiar and will, I hope, elicit a very free discussion. I will take it for granted that you are familiar with the histology and pathology of tubercle, subjects too well known to permit of discussion, and will pass at once to the consideration of the predisposing causes, the symptoms and treatment of pulmonary tuberculosis.

Prominent among the predisposing causes must be placed the hereditary tendency to the disease. It is not to be understood that the disease itself is inherited, but that the patient inherits from the parent pulmonary tissue, which has insufficient resisting power to repel the invasion of the bacilli. It may be well to remember that in a disease so prevalent as the one under consideration that the atmosphere, under favorable conditions, is frequently contaminated with the tubercle bacilli or their spores, chiefly as the result of the drying of tubercular sputa, and it must happen that every individual, or at least those living in communities where tuberculosis exists, is frequently exposed to the contagium of the disease. If we also bear in remembrance that there is a standing declaration of war to the death between the bacilli and the tissues, and that in the conflict the weakest must succumb, we will readily realize why a parent with diseased lungs may bequeath to his posterity a legacy of tuberculosis. Another potent cause of pulmonary tuberculosis is prolonged contact with the contagium of the disease, as may be witnessed where the husband whose wife is tubercular contracts the disease, or *vice versa;* or in the melancholy but numerous instances where a member of a family without a tubercular ancestry contracts the disease, and the remaining members who have been in intimate contact with the patient are subsequently attacked.

Any condition or disease which lowers a patient's vitality is a predisposing cause of pulmonary tuberculosis, and it is the more powerful if the condition directly affects the respiratory apparatus. Among such may be mentioned imperfect development of the chest, occupations which hinder free and full respiratory movements, bronchitis, pleuritis and pneumonitis, the latter especially if the inflammatory exudate is slow in undergoing resolution.

It is unnecessary to pursue this part of the subject further, and I will only emphasize what my remarks must have indicated, that tuberculosis is a contagious disease, but in order that the contagium may thrive there must be conditions in the soil on which it is planted that are favorable to its growth.

When pulmonary tuberculosis has reached an advanced stage its manifestations are so plain, that he who runs may read; there is something so characteristic in the combination of symptoms that go to form the make up of such a case, that a diagnosis is already made before the patient has been examined. The emaciated figure, the stooped shoulders, the sunken chest, the rapid breathing, the hacking cough and the clubbed fingers are so unmistakable and so familiar that even the laity are seldom in error in their conclusions. But of such cases I have little to say; they have probably passed the point where we can aid them; their fate is already sealed, and the utmost we can do is to ward off a little longer the impending doom, and ease the rugged pathway along which the victims of this cruel and relentless disease must tread, until death, less merciless, stills the hacking cough and in eternal sleep throws the mantle of oblivion over the scene.

Of much greater importance to us and to the patient is the diagnosis of the disease in the early stages, important because the earlier the disease is recognized the easier is it to be contended with, and the more beneficent is our therapeutics.

Of such importance do I consider the early diagnosis of phthisis that I feel constrained, in order to emphasize its importance, to digress from the subject, and speak a few words on the curability of phthisis. Consumption is usually regarded, by the laity, as invariably a fatal disease, and I fancy, also, by some of our professional brethren, especially that class not yet extinct, who have a contemptuous disregard for physical diagnosis and microscopical achievements. And they have good reasons for such judgment, since the disease is seldom recognized by them until it has reached a stage where its progress cannot be retarded, nor a fatal termination averted. That consumption is necessarily a fatal disease is a false and grievous conception, and the sooner it is totally eradicated the better will it be for the profession and humanity.

Consumption is often cured when in the incipient stage. This fact was early impressed on me by numerous illustrations which I witnessed a few years ago among the river men while under treatment in the marine wards of Mercy Hospital. These men are notoriously careless of their physical welfare, and their occupations are of such a nature that pulmonary complaints are rife among them. At the time I referred to I was anxious to familiarize myself with the early symptoms of phthisis, and made the routine habit of examining every marine's lungs, regardless of the disease for which he was admitted. I was astonished at the number of cases of incipient phthisis that were discovered, often among those who did not complain of any pulmonary trouble, or, at the most, of a slight cough. It was even more astonishing to witness the frequency with which the physical signs of incipient phthisis would disappear under improved hygienic conditions and appropriate treatment. It is only fair to add that in these cases the diagnosis was based on the physical signs, as microscopical examination of the sputa was not made. Since that time I have witnessed a number of similar cases, although such a happy termination does not always take place.

I do not put before you my own limited experience as conclusive proof, but will bring forward the cold facts as gleaned from a source into which mistakes in diagnosis and results are not liable to creep, namely: *post mortem* examinations. The following is quoted from John Hughes Bennett's article on phthisis, in Reynold's System of Medicine (Vol. II., page 130): "The careful *post mortem* examinations now made with such regularity in our large hospitals have demonstrated the frequent occurrence of old condensations, cicatrices, and calcareous concretions at the apices of the lungs in persons of advanced age who have died of other diseases. In 1845 I pointed out that in the Royal Infirmary of Edinburgh they occurred in the proportion of from one-fourth to one-third of all the individuals who died after the age of forty. Roger and Boudet had previously shown that at the Salpetriere and Bicetre Hospitals in Paris, among individuals above the age of seventy, they occurred in one-half and in four-fifths of the cases respectively. There can be no doubt that these cicatrices and concretions indicate the healing and drying up of cavities and softened tubercular matter at some previous period in the life of the individual, and the consequent spontaneous cure of the disease in a considerable number of persons."

I return to the diagnosis of phthisis in its incipient stage, the importance of which I endeavored to impress upon you by pointing out that in this stage the disease, under proper hygienic and therapeutic treatment, was frequently curable.

Phthisis, in its incipient stage, is usually a very insidious disease. There is no line of demarcation to distinguish when health ended and disease began. Perhaps the very first indication is a condition of malaise—an indefinable feeling of not being well, without any concomitant symptoms to indicate pulmonary mischief. Often there is tenderness over one or both the apices of the lungs, or fleeting pains may attract the patient's attention to this region. A slight cough, associated with but little expectoration, is a usual concomitant, although the cough may be severe and the expectoration profuse where a bronchitis has been an accompaniment or predisposing factor in the causation of the tuberculosis.

Another symptom of considerable significance is a slight elevation of temperature, which may not be over half a degree. If the temperature should be elevated on several successive examinations, and if there is no obvious cause for it, the presence of tuberculosis should be strongly suspected, and a careful examination of the lungs should be made. As regards the methods of examining the lungs a few words may be said. The patient should be stripped, only the slightest covering to the chest, such as light underclothing, is admissible. This rule should be rigorously observed, regardless of whether the patient be male or female, child or adult. If the patient, through false ideas of modesty, should object, it is the physician's duty to refuse to stake his reputation on a hazardous guess as to the condition of patient's lungs, whose sounds are muffled from his ear by the interposition of a pair of corsets, and the various other paraphernalia which deck the female form.

The physician who, under such circumstances, ventures to give an opinion as to the presence or absence of incipient phthisis, must either be gifted with an abnormal acuteness in hearing and discrimination in eliminating the extraneous sounds produced by the respiratory movements on the wearing apparel, or, what is more likely, he has a firm conviction that if he did hear the pulmonary sounds he would be unable to tell what they meant, and very considerately draws the line on his imposition at the point which necessitates the removal of the patient's garments.

To my mind the most characteristic of the physical signs of incipient phthisis is the broncho-vesicular breathing heard usually at the apex of the lung, and often best heard at the supraspinous fossa. The prolonged expiration heard in this region is significant, but care must be exercised that the blowing sound of the large air tubes be eliminated. With the broncho-vesicular breathing there is often associated a few crackling râles and a jerking inspiration, although the latter may be present when disease is absent. Percussion dullness and increased vocal fremitus are of very great weight as corroborative evidence of tuberculosis ; but the inability to detect either is of very little negative value.

Another very important element in the diagnosis is the microscopical examination of the sputa; and here we come to the only pathognomonic symptom of incipient phthisis, and that is the demonstration in the sputa of the tubercle bacilli. The negative value of the absence of the bacilli is of but little weight, except on repeated examination of the sputa.

I will pass over the methods of staining and examination for bacilli ; also the symptoms of advanced pulmonary consolidation, and the destruction of lung tissue with formation of cavities, and hasten to the no less important subject of treatment.

Prominent among the remedial agencies must be placed improved hygienic surroundings, a generous diet, and suitable climatic changes. A climate which permits of out-door exercise the greatest number of days in the year is the one to which, in a general way, preference should be given. Where there is a tendency to hemorrhages an elevated climate should be avoided, but under other circumstances an elevated climate, such as Colorado affords, is preferable, inasmuch as the rarefaction of the atmosphere necessitates a fuller expansion of the lungs, which, probably, tends to the re-opening of the closed air cells. It may be well to sound a note of warning at this point on the folly of sending patients to another climate for the winter, in the hope that a permanent benefit will ensue. It seems to be a settled fact that patients who have been benefited by such a change are under the necessity of remaining in the advantageous climate, as their return, except for a limited period, is fraught with the danger of a renewed outbreak of the disease.

Among the therapeutic agents may be mentioned cod liver oil, which may be said to be of value only in those comparatively few cases where it does not nauseate. The hypophosphites are doubtless of value, as are a score of other remedies. A favorite treatment of mine, and one which was chiefly used in the cases of the marines already mentioned, is the long continued use of the following prescription:

| | | |
|---|---|---|
| ℞. Hyd. chl. corr., | . . | gr. j. |
| Ammon. chlorid., | . . | ʒ ji. |
| Potas. iodidi., | . . | ʒ jss. |
| Syr. pruni Virg., | . . | ℥ iv. |

M. Sig. One teaspoonful after meals and at bedtime.

The most remarkable remedy yet brought to light, and one which has made its already famous discoverer a household name in every home in the land, is Koch's lymph, or as it is more properly called, "tuberculin."

This remedy is the product of the tubercle bacilli. It is a brownish colored liquid, is exceedingly powerful, and is only used in the dilute form by hypodermatic injection, being inert if taken by the stomach. Now that the excitement following the announcement of the discovery has subsided and a considerable time has elapsed in which to test the value of the remedy, we may venture to give an opinion as to its merits and mode of action.

In order to understand the explanation given by Koch regarding the latter, it is necessary to speak a few words on the phenomena of coagulation necrosis, which is a peculiar change resembling coagulation caused by the action on the cells of a chemical product, or ptomaine, produced by bacterial agencies. It is supposed that the caseation of a tubercular nodule is a condition of coagulation necrosis produced by the action on the tubercle cells of a ptomaine, the result of the growth of the tubercle bacilli. Koch reasoned that if he could add to the already ptomaine poisoned tubercular tissue sufficient of the same poison he could produce at will a coagulation necrosis of the living tubercular tissue, and thus place it in a condition to be either thrown off from the surface or removed by absorption. That this is the correct theory of the action of tuberculin I do not venture to express an opinion, but it explains in a satisfactory manner some of the phenomena observed in its use, such as the curious fact that the tubercle bacilli are not killed by the use of tuberculin, and that only living tubercular tissue is affected.

It is exceedingly difficult to give a satisfactory opinion as to the value of a remedy like this which has so many biased opponents and blindly enthusiastic advocates. What I have to say is the result of considerable attention to the voluminous literature of the subject, as well as personal observation of a great many cases under treatment in the hospital of Berlin, and also about three months' experience with the use of the remedy in this city.

In order to be brief, my opinions must necessarily be dogmatical.

Tuberculin is of considerable value as a diagnostic agent but is not always reliable, the reaction being often wanting in the surgical forms of tuberculosis and also occasionally in the pulmonary form.

As a rule the most marked benefits are obtained where the necrotic tubercular tissue can be thrown off from the surface as in lupus, tubercular laryngitis and tubercular ulcers.

Most cases of pulmonary tuberculosis in the early stages will be benefited by the remedy, some to a very marked degree, so that a cure may be fairly claimed. Advanced cases of pulmonary tuberculosis where cavities have formed, and especially if the disease is rapid in its progress, are not likely to be benefited, and will probably be injured by the treatment.

In the surgical forms of tuberculosis, tuberculin will be an aid to the operative treatment by assisting in the complete eradication of the tubercular tissue.

The nature of this paper restricts it within narrow limits. Many points upon which I would like to touch must be passed unnoticed, but I hope that this fact may, of itself, stimulate among the members a friendly criticism of the paper and a free discussion of tuberculosis.

